# INCB054828 (pemigatinib), a potent and selective inhibitor of fibroblast growth factor receptors 1, 2, and 3, displays activity against genetically defined tumor models

**DOI:** 10.1371/journal.pone.0231877

**Published:** 2020-04-21

**Authors:** Phillip C. C. Liu, Holly Koblish, Liangxing Wu, Kevin Bowman, Sharon Diamond, Darlise DiMatteo, Yue Zhang, Michael Hansbury, Mark Rupar, Xiaoming Wen, Paul Collier, Patricia Feldman, Ronald Klabe, Krista A. Burke, Maxim Soloviev, Christine Gardiner, Xin He, Alla Volgina, Maryanne Covington, Bruce Ruggeri, Richard Wynn, Timothy C. Burn, Peggy Scherle, Swamy Yeleswaram, Wenqing Yao, Reid Huber, Gregory Hollis

**Affiliations:** 1 Discovery Biology, Incyte Research Institute, Wilmington, Delaware, United States of America; 2 Discovery Chemistry, Incyte Research Institute, Wilmington, Delaware, United States of America; Florida International University, UNITED STATES

## Abstract

Alterations in fibroblast growth factor receptor (FGFR) genes have been identified as potential driver oncogenes. Pharmacological targeting of FGFRs may therefore provide therapeutic benefit to selected cancer patients, and proof-of-concept has been established in early clinical trials of FGFR inhibitors. Here, we present the molecular structure and preclinical characterization of INCB054828 (pemigatinib), a novel, selective inhibitor of FGFR 1, 2, and 3, currently in phase 2 clinical trials. INCB054828 pharmacokinetics and pharmacodynamics were investigated using cell lines and tumor models, and the antitumor effect of oral INCB054828 was investigated using xenograft tumor models with genetic alterations in FGFR1, 2, or 3. Enzymatic assays with recombinant human FGFR kinases showed potent inhibition of FGFR1, 2, and 3 by INCB054828 (half maximal inhibitory concentration [IC_50_] 0.4, 0.5, and 1.0 nM, respectively) with weaker activity against FGFR4 (IC_50_ 30 nM). INCB054828 selectively inhibited growth of tumor cell lines with activation of FGFR signaling compared with cell lines lacking FGFR aberrations. The preclinical pharmacokinetic profile suggests target inhibition is achievable by INCB054828 in vivo with low oral doses. INCB054828 suppressed the growth of xenografted tumor models with FGFR1, 2, or 3 alterations as monotherapy, and the combination of INCB054828 with cisplatin provided significant benefit over either single agent, with an acceptable tolerability. The preclinical data presented for INCB054828, together with preliminary clinical observations, support continued investigation in patients with FGFR alterations, such as fusions and activating mutations.

## Introduction

Deregulation of receptor tyrosine kinase (RTK) signaling has the potential to promote the acquisition of several hallmarks of cancer cells notably self-sustaining proliferation, enhanced angiogenesis, evasion of cell death, and increased migration and invasion [1, 2]. Aberrant activation of RTK signaling can proceed through conserved mechanisms including genomic amplification and protein overexpression, gain-of-function mutations, chromosomal translocations, and autocrine activation [3]. Through systematic sequencing of cancer genomes, genetic alterations in many RTKs were identified across numerous tumor types [4]. The oncogenic activity of deregulated RTKs (e.g. ABL, EGFR, and ALK) is well documented, and selective small-molecule inhibitors have been approved for treatment of biomarker-defined patients [5].

The mammalian fibroblast growth factor receptor (FGFR) family is composed of 4 highly-conserved receptors (FGFR1, FGFR2, FGFR3, and FGFR4) with an extracellular ligand-binding domain, a single transmembrane domain, and an intracellular tyrosine kinase domain [6]. Among 22 FGF ligands, 18 bind to FGFRs leading to dimerization, activation of the kinase domain, and transphosphorylation of receptors [7]. Signal transduction occurs through phosphorylation of substrate proteins (e.g. FGFR substrate 2) that leads to activation of the RAS-MAP kinase and PI3 kinase-AKT pathways and phospholipase Cγ that activates the protein kinase C pathway. In some cellular contexts, signal transducer and activator of transcription (STAT) proteins are also activated by FGFRs. In many cases, FGFR pathway activation promotes cell proliferation, survival, and migration; however, cellular context plays an important role, and in certain tissues, FGFR signaling results in growth arrest and cellular differentiation [6, 8, 9].

There is strong genetic and functional evidence that dysregulation of FGFR can lead to the establishment and progression of cancer. Genetic alterations in FGFR1, FGFR2, and FGFR3 are reported in many tumor types and include all of the mechanisms described leading to constitutive activation of the receptors or aberrant ligand-dependent signaling through FGFRs [8, 10]. In most cases, the alterations do not change the protein sequence of the active kinase domain. In a pan-cancer survey of >4,800 solid tumors, 7.1% had alterations in FGFR genes with wide distribution and variable frequency; however, enrichment was observed in several cancers including bladder, breast, and lung, consistent with previous tumor-specific studies [11–16]. Among these aberrations, chromosomal translocations that deregulate kinase activity are strong oncogenic drivers, and fusions involving FGFR1, FGFR2, and FGFR3 have been described [17]. Recurrent intrachromosomal fusions between FGFR1 and TACC1, and FGFR3 and TACC3 occur in approximately 3% of glioblastoma multiforme and 4% of bladder cancer, respectively [18, 19]. In intrahepatic cholangiocarcinoma, fusions of FGFR2 with various partners are reported in 10–15% of cases [20, 21]. Similarly, FGFR1 is translocated to multiple chromosomal loci in a rare myeloproliferative neoplasm: myeloid/lymphoid neoplasms with FGFR1 rearrangement [22, 23]. Evidence suggests that the genetically activated FGFR pathway sensitizes FGFR-altered cancer cells to knockdown or inhibition of these receptors [16, 24–30]. Collectively, these data indicate that alterations in FGFR genes can be driver oncogenes in some, though not all, contexts. Therefore, pharmacological targeting of FGFRs may provide therapeutic benefit to selected cancer patients; indeed, early clinical trials of FGFR inhibitors for several indications have provided proof-of-concept [31, 32].

We present the molecular structure and preclinical characterization of INCB054828 (pemigatinib), a novel, selective inhibitor of FGFR 1, 2, and 3. INCB054828 differs from earlier kinase inhibitors with FGFR activity with high selectivity for FGFR family members; its potency and drug-like properties have translated to antitumor efficacy at very low doses in preclinical models. In phase 1 clinical trials, INCB054828 exhibited a favorable safety profile and target inhibition based on pharmacodynamic markers of FGFR inhibition [33]. INCB054828 is currently being investigated in 3 phase 2 clinical trials in genetically-selected patients with urothelial carcinoma, cholangiocarcinoma, and 8p11 myeloproliferative syndrome.

## Materials and methods

### Cell lines and reagents

All cell lines were purchased directly from ATCC^®^ (Rockville, MD. Catalogue numbers: NCI-H1581, CCL-5878; DMS-114, CRL-2066; KG1a, CCL-246.1; KATO III, HTB-103; AN3CA, HTB-111), DSMZ (Braunschweig, Germany. Catalogue numbers: Ba/F3, ACC-300; RT-112, ACC-418; RT-4, ACC-412; OPM-2, ACC-50), the Japanese Collection of Research Bioresources Cell Bank (Osaka, Japan. Catalogue number: KMS-11, JCRB1179), or Lonza (Basel, Switzerland. Catalogue number: HUVEC, C-2519A), and were acquired between 2003 and 2013. All cell lines were confirmed to be negative for mycoplasma (Bionique Testing Laboratories, Inc., Saranac Lake, NY), and cell lines used for in vivo testing were authenticated by short tandem repeat analysis between 2012 and 2017. INCB054828 was synthesized at Incyte Corporation (Wilmington, DE).

### Western blotting and ELISA

All antibodies were purchased from commercial sources as follows: antibodies against FGFR1 (monoclonal [rabbit], catalogue no. 9740, Antibody Registry Identifier AB_11178519), ERK (monoclonal [mouse], 9107, AB_10695739), phospho-p42/44 ERK1/2 (monoclonal [rabbit], 4370, AB_2315112), phospho-STAT5 (Tyr694; monoclonal [rabbit], 4322, AB_10544692), and STAT5 (monoclonal [rabbit], 9358, AB_659905) were from Cell Signaling Technologies (Danvers, MA) and used at 1:1000 dilution; FGFR3 (monoclonal [rabbit], AB133644, AB_2810262) and FRS2 (polyclonal [rabbit], AB10425, AB_2247176) were from Abcam (Cambridge, UK) and used at 1:2000; and phospho-FRS2 (Tyr436; polyclonal [rabbit], AF5126, AB_2106234) was from R&D Systems (Minneapolis, MN) and used at 1 μg/mL. The pFGFR2 enzyme-linked immunosorbent assay (ELISA) kit was purchased from R&D Systems and ELISA was performed per manufacturer protocol.

### Cell viability assay

Cell viability assays were performed after 72 hours of incubation with a serial dilution of INCB054828 or 0.1% dimethyl sulfoxide as control using the CellTiter-Glo^®^ ATP assay (Promega, Madison, WI). All cell lines were tested with a minimum of 3 independent experiments, and data are reported as the mean ± standard deviation (S.D.).

### Proximity ligation assay

In situ phosphorylation of FGFR3 on RT-4 and RT-112 cells was performed using the Duolink PLA^®^ (Proximity Ligation Assay, Sigma Chemical, St. Louis, MO) with the following primary antibodies: rabbit anti-human FGFR3 (Abcam) and mouse anti-human pFGFR (Y653/654) (Cell Signaling) and the Duolink In Situ PLA^®^ Probe anti-rabbit PLUS and Duolink In Situ PLA^®^ Probe anti-mouse MINUS secondary probes.

### In vivo experiments

Non-Good Laboratory Practice studies intended to characterize the pharmacology of INCB054828 were conducted in accordance with Incyte Corporation's Animal Use Protocols and DuPont Stine-Haskell standard operating procedures, and the study protocol was approved by the Haskell Animal Welfare Committee (Protocol: HAWC008). Animals were housed in barrier facilities fully accredited by the Association for Assessment and Accreditation of Laboratory Animal Care, International. All of the procedures were conducted under the supervision of a veterinarian and in accordance with the U.S. Public Health Service Policy on Humane Care and Use of Laboratory Animals. In accordance with the study protocol, animals were humanely euthanized (by carbon dioxide exposure) if they showed severe signs of pain or distress, if there was evidence of tumor necrosis or ulceration, if tumor growth impeded movement, if tumor weight exceeded 10% of body weight for 2 consecutive measurements, or if body weight loss exceeded 20% of baseline values. Analgesics and anesthetics could be used to minimize animal suffering and distress; neither were required during these studies. Animals in the study were monitored twice daily and all dose levels presented were well tolerated with no unexpected drug-related deaths. For efficacy experiments, tumor volumes were allowed to reach the point of appropriate randomization for each model, and tumors were measured and analyzed for adequate shape and placement. The mice with acceptable tumors were randomized using a serpentine method and individual mice were exchanged (if needed) to allow for the tightest possible starting groups. Tumor volume calculation followed the method described by the Jackson Laboratory (http://tumor.informatics.jax.org/mtbwi/live/www/html/SOCHelp.html).

For the KATO III model, female severe combined immunodeficiency (scid) mice (5–8 weeks of age; Charles River Laboratories, Wilmington, MA) were inoculated with KATO III tumor brei 1:10 (w/v). The tumor brei was prepared from several donor mice and implanted subcutaneously on the flank of the mice in a 1:1 (v/v) mixture of HEPES-buffered saline solution and matrigel (BD Biosciences, Billerica, MA #354248) of 0.2 mL. The treatment of tumor bearing mice was started 9 days after tumor inoculation for efficacy studies and 17–20 days for pharmacodynamic studies. Starting mean tumor volume in all groups was 281 mm^3^ and groups consisted of 8 animals. For pharmacodynamic studies, groups of 3–5 animals were included in the study with average tumor sizes of 334–548 mm^3^.

For the RT112 model, female Rowett Nude rats (5–8 weeks of age, Charles River Laboratories) were inoculated with 5 × 10^6^ RT112 tumor cells in phosphate buffered saline (PBS) mixed 1:1 (v/v) with matrigel (BD Biosciences #354234). The inoculation of 0.5 mL was performed subcutaneously on the flank. The treatment of tumor bearing rats was started 8 days after tumor inoculation. Starting mean tumor volume in all groups was approximately 335 mm^3^ and groups consisted of 8 animals.

For the H1581 model, female nu/nu mice (6–11 weeks of age, Charles River Laboratories) were inoculated with 5 × 10^6^ tumor H1581 cells in PBS mixed 1:1 (v/v) with matrigel (BD Biosciences #354234). The inoculation was performed subcutaneously on the flank. The treatment of tumor bearing mice was started 12 days after tumor inoculation. Starting mean tumor volume in all groups was approximately 270 mm^3^ and groups consisted of 7 animals.

For the cholangiocarcinoma patient derived xenograft (PDX) model, CTG-0997 tumor fragments (Champions Oncology, Baltimore, MD) were implanted into the left flank of nu/nu mice (5–8 weeks of weeks of age, Harlan Laboratories, Indianapolis, IN). The treatment of tumor bearing mice was started when the mean tumor volume was approximately 188 mm^3^ and groups consisted of 12 animals.

For the KG1 model, female NOD scid gamma (NSG) mice engrafted with CD34+ umbilical cord blood cells (26 weeks of age, Jackson Laboratories, Bar Harbor, ME) were inoculated with 1 × 10^7^ tumor KG1 cells in PBS mixed 1:1 (v/v) with matrigel (VWR, Radnor, PA). The inoculation was performed subcutaneously on the right flank. The treatment of tumor bearing mice was started 7 days after tumor inoculation. Starting mean tumor volume in all groups was approximately 350 mm^3^ and groups consisted of 6 animals.

For all studies, experimental therapeutic agent, INCB054828, was administered to mice orally. Tumor volume was calculated in 2 dimensions using the equation: volume = [length × (width^2^)] / 2, where the larger number was length and the smaller number was the width. Effects on tumor growth were reported as percent tumor growth inhibition calculated as [1 –(treatment volume / control volume)] × 100, where control volume was the vehicle/untreated tumor volume on a given day and treatment volume was any treatment group tumor volume on that same day. Statistical significance of differences between treatment and vehicle controls was assessed using analysis of variance single factor test, unless otherwise noted.

### Pharmacodynamic studies

Female C57BL/6 mice, 5–8 weeks of age, were obtained from Charles River Laboratories. A single dose of INCB054828 was administered to mice at 10 mL/kg, and mice were fasted overnight before collection of samples. Twenty-four hours after dosing, mice were anesthetized under isoflurane. Approximately 200 μL of whole blood was collected by capillary retro-orbital sinus sampling into lithium heparin collection tubes (RAM Scientific, Yonkers, NY). Blood was maintained at 4°C and transported for analysis to DuPont Haskell Global Centers for Health and Environmental Science (Newark, DE). Each sample was analyzed for levels of inorganic phosphate within 4 hours of receipt. Serum clinical chemistry parameters were determined using an Olympus^®^ AU640 clinical chemistry analyzer (Beckman Coulter, Brea, CA). Both phosphate and calcium are manufacturer-supplied reagents and methods.

Please see S1 Appendix for details of the **Enzyme selectivity panel**; **Receptor tyrosine kinase IC**_**50**_ **inhibition assay**; **Absorption, distribution, metabolism and excretion assays**; and **Pharmacokinetic analyses** methods.

## Results

### INCB054828 is a potent and selective inhibitor of FGFRs 1, 2, and 3

**Fig 1:**
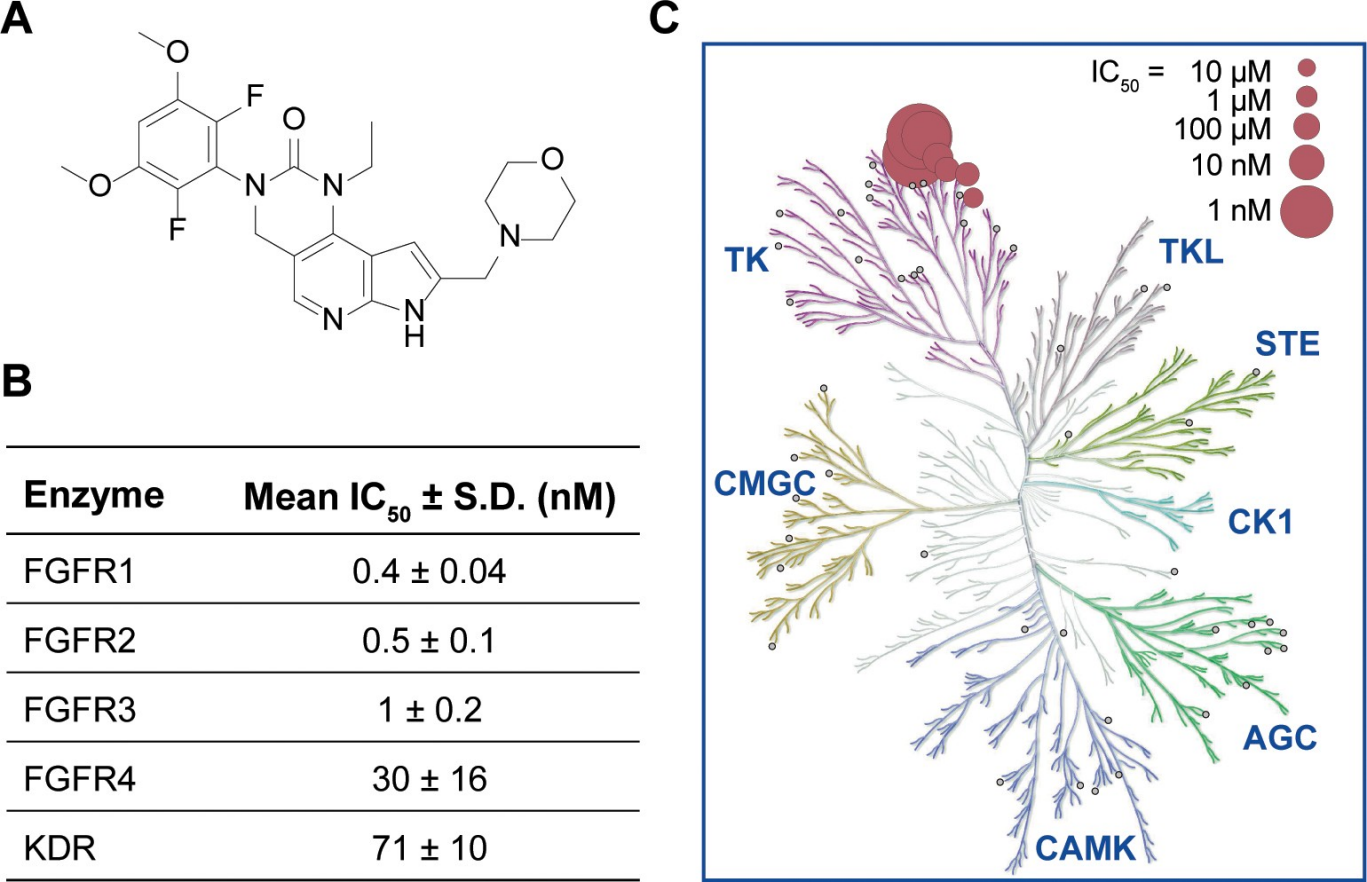
Molecular structure and In Vitro profile of INCB054828. (A) Molecular structure of INCB054828. (B) Potency of INCB054828 against FGFR kinases. Activity of recombinant human enzymes was assayed as described at the Michaelis–Menten constant (Km) ATP for each enzyme. The mean IC_50_ and S.D. for 6 (FGFR4) or 8 (FGFR1, FGFR2, FGFR3, KDR) independent experiments using multiple lots of inhibitor is reported. (C) Selectivity profile of INCB054828. Biochemical IC_50_ values of INCB054828 for 56 kinases. Small gray circles indicate tested kinases with IC_50_ >10,000 nM. Among non-FGFRs, KDR and c-KIT were the only kinases inhibited with an IC_50_ value <1,000 nM.

The chemical structure of INCB054828, an ATP-competitive inhibitor of the FGFR enzymes, is shown in Fig 1A. Enzymatic assays with recombinant human FGFR kinases showed potent inhibition of FGFR1, FGFR2, and FGFR3 by INCB054828 (half maximal inhibitory concentration [IC_50_] values of 0.4, 0.5, and 1 nmol/L, respectively) with weaker activity against the related family member FGFR4 (Fig 1B). Consistent with an ATP-competitive binding mode, the FGFR1 IC_50_ of INCB054828 shifted from 0.6 to 6 nM when the ATP concentration in the enzyme assay was increased from 50 to 5,000 μM (S1 Fig). The inhibitory activity was reversible upon dilution of the reaction (S1 Fig). The selectivity of INCB054828 was determined by assaying a panel of 56 diverse tyrosine and serine-threonine kinases under high throughput conditions using a fixed ATP concentration of 1 mM for all reactions. Only vascular endothelial growth factor receptor-2/kinase insert domain containing receptor (VEGFR2/KDR) (IC_50_, 182 nM) and c-KIT (IC_50_, 266 nM) showed IC_50_ values less than 1,000 nM indicating that INCB054828 exhibited high selectivity for the FGFR kinases (Fig 1C, S1 Table). A cellular growth assay using human umbilical vein endothelial cells showed greater than 80-fold difference in the potency of INCB054828 to inhibit FGF-dependent growth compared with VEGF-dependent growth (S2 Fig). Subsequent profiling against a broader panel of 161 kinases (PerkinElmer, Akron, OH) confirmed the selectivity; no additional kinases were significantly inhibited by INCB054828 at a concentration of 100 nM besides the 4 FGFR enzymes and VEGFR2 (S2 Table).

To confirm that the potency of INCB054828 was not related to recombinant expression of the tagged fusion proteins or of the assay format, phospho-FGFR2 was assessed in the KATO III cell line that expresses high levels of wild-type FGFR2. A quantitative ELISA assay showed inhibition of FGFR2 phosphorylation (IC_50_ = 3 nM; S3 Fig [A]). To correct for human protein binding, the KATO III cell line was spiked into normal donor blood with serial dilutions of the inhibitor, and the IC_50_ for inhibition of phospho-FGFR2 levels was determined to be 10.9 nM (S3 Fig [B]). The ability of INCB054828 to inhibit autophosphorylation of FGFR was further tested using Ba/F3 cell lines engineered to express the kinase domains of FGFR as fusions with the dimerization domain of ETV6 (TEL); the results showed balanced inhibition with IC_50_ values of 3 and 4 nM, for FGFR1 and FGFR3, respectively (S3 Fig [C+D]).

**Fig 2:**
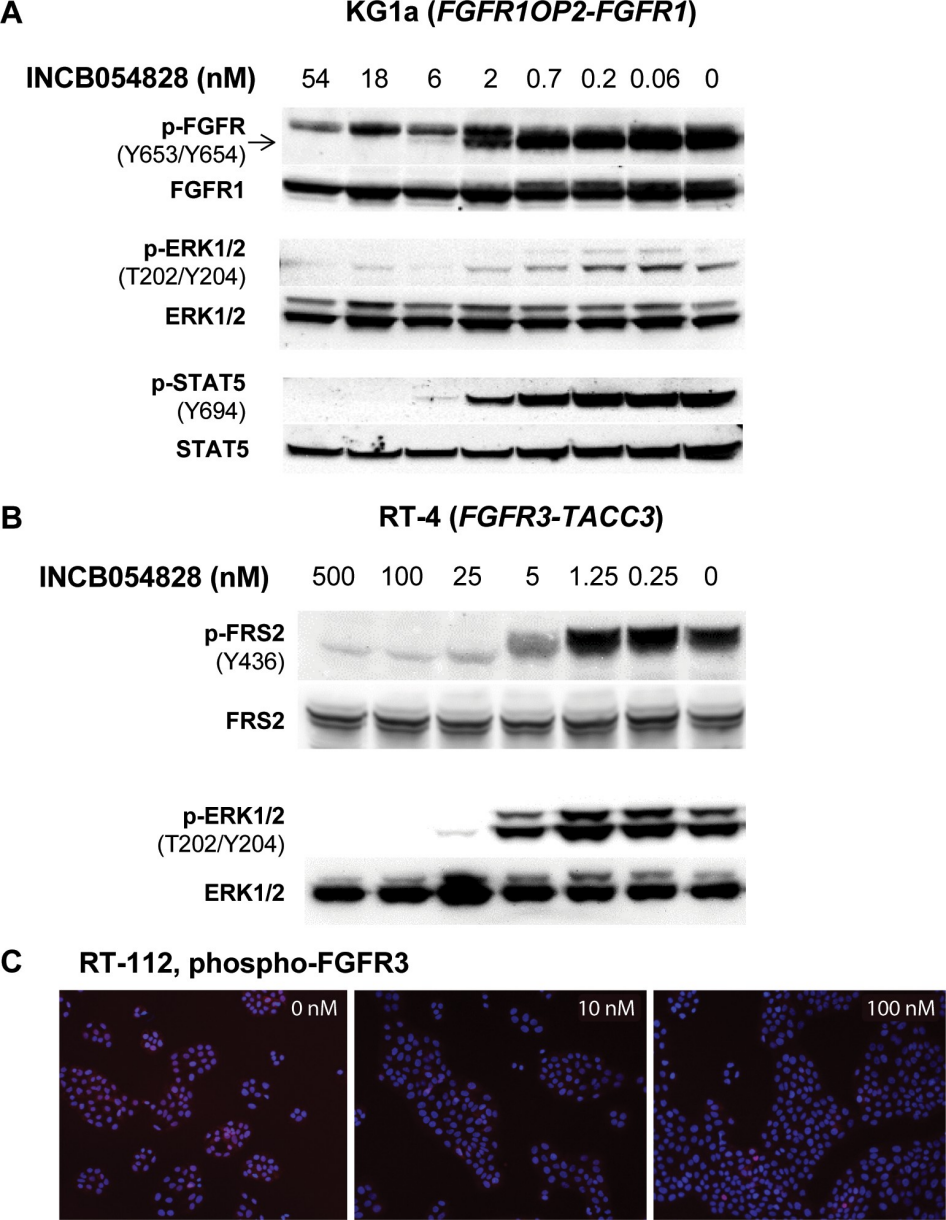
INCB054828 inhibits FGFR-dependent signaling pathways. (A) KG1a or (B) RT-4 cells were treated with INCB054828 for 2 hours, lysed and subjected to immunoblotting for phospho- and total proteins in the FGFR signal transduction pathway including FGFR, ERK, FRS2, and STAT5. (C) Concentration-dependent inhibition of phospho-FGFR3 by INCB054828 in RT-112 cells was determined using a proximity ligation assay with a mouse monoclonal anti-phospho-FGFR (Y653/Y654) and rabbit anti-FGFR. Original Western blot images are shown in S1 File (S1 Raw images).

To investigate the effect of INCB054828 treatment on intracellular signaling, we used Western blot analysis for markers of FGFR pathway activation. We chose 2 cell lines derived from malignancies associated with genetically activated FGFR1 (KG1a, 8p11-positive acute myeloid leukemia [AML]) and FGFR3 (RT-4, urothelial carcinoma) that are also indications in which INCB054828 is currently being investigated in phase 2 clinical trials. In the KG1a cell line that bears an *FGFR1OP2*-*FGFR1* translocation, a concentration of greater than 5 nM reduced levels of phospho-FGFR to basal levels (Fig 2A). Phospho-ERK and phospho-STAT5 are also reduced with the same concentration dependence, consistent with potent suppression of FGFR activation by the inhibitor. Treatment of the bladder cancer line RT-4 that harbors an *FGFR3-TACC3* translocation [19] with INCB054828 strongly suppresses levels of phospho-FRS2, a scaffolding protein that is a substrate of FGFR, and phospho-ERK (Fig 2B). FGFR3 phosphorylation was not detectable by Western blotting; however, a decrease was detected by proximity ligation assay that uses polymerase chain reaction to amplify the signal from the bound antibodies to phospho- and total FGFR3 (S4 Fig). Using this method, potent inhibition of FGFR3 by INCB054828 (<10 nM) was confirmed in a second urothelial cell line RT-112 that also harbors the FGFR3-TACC3 fusion (Fig 2C).

INCB054828 selectively inhibits the growth of tumor cell lines with activation of FGFR signaling (Table 1). The most sensitive lines had GI_50_ values (concentration required to inhibit growth by 50%) less than 15 nM. In comparison, the GI_50_ values for a panel of hematologic and solid tumor cell lines that lacked known alterations in the FGFR genes exceeded 2,500 nM (S3 Table); many of these cell lines are known to have dependencies on other oncogenes (e.g. EGFR, HCC-422; K-Ras, A549, and UMUC3). The data reveal a clear separation in sensitivity to INCB054828 between cell lines with genetic alterations in FGFR1, FGFR2, or FGFR3 and cell lines lacking these aberrations. Furthermore, there was no inhibition of the proliferation of primary T cells from normal donors up to 1,500 nM (S5 Fig).

**Table 1:** Growth inhibition of tumor cell lines with activation of FGFR signaling by INCB054828.

Cell Line (Histology)	FGFR Alteration	Mean GI_50_ ± S.D. (nM)
**H1581 (lung cancer)**	FGFR1 amplification	14 ± 9
**DMS-114 (lung cancer)**	FGFR1 amplification	27
**KG1a (AML)**	FGFR1OP2-FGFR1 fusion	3 ± 1
**KATO III (gastric cancer)**	FGFR2 amplification	3 ± 1
**AN3CA (endometrial cancer)**	FGFR2 N310R/N549K mutations	48 ± 28
**RT-112 (bladder cancer)**	FGFR3-TACC3 fusion	7 ± 3
**RT-4 (bladder cancer)**	FGFR3-TACC3 fusion	12
**KMS-11 (myeloma)**	IgH-FGFR3 translocation	362 ± 282
**OPM-2 (myeloma)**	IgH-FGFR3 translocation	18 ± 9
**Engineered Cell Lines**		
**Ba/F3-FGFR1-ZFN298**	FGFR1 fusion, 8P11 MPN	0.9 ± 0.4
**Ba/F3-FGFR2-CCDC6**	FGFR2 fusion, cholangiocarcinoma	1.2 ± 0.2
**Ba/F3-FGFR2-AHCYL**	FGFR2 fusion, cholangiocarcinoma	1.1 ± 0.3

MPN, myeloproliferative neoplasm

### Pharmacokinetic profile of INCB054828

In vitro absorption, distribution, metabolism, and excretion (ADME) properties and pharmacokinetic parameters of INCB054828 were also determined (Table 2). In vitro, INCB054828 showed moderate to high permeability across Caco-2 cell monolayers, exhibited moderate metabolic stability in human liver microsome, and demonstrated IC_50_s greater than 25 μM for all of the CYP isoforms tested (1A2, 2B6, 2C8, 2C9, 2C19, 2D6, and 3A4). The precise IC_50_ for the inhibitory effect of INCB054828 on hERG potassium current was not calculated due to compound insolubility, but was estimated to be >8 μM (Data provided by ChanTest Corporation, Cleveland, OH).

**Table 2:** In vitro ADME and pharmacokinetics of INCB054828.

**In Vitro ADME**			
Caco-2 (P_app_ 10^−6^ cm/sec; A–B at 50 μM)	11		
Intrinsic clearance (L/h/kg)^a^	0.8		
PPB (% free; rat, monkey, human at 1 μM)	3.0, 8.2, 11.3		
CYP3A4 inhibition (IC_50_, μM)	>25		
**Pharmacokinetics**^**b**^			
	**Rat**	**Dog**	**Monkey**
CL (L/h/kg)^c^	1.03	0.183	0.198
Hepatic ER (%)^c^	31	10	8
Vss (L/kg)^c^	1.85	3.49	0.584
t_½_(h)^c^	4.0	15.7	10.3
C_max_ (μM)^d^	2.26	1.77	0.766
AUC (μM*h)^d^	7.67	22.1	6.19
%F^d^	>100	98	29

^a^Study conducted with human liver microsomes.

^b^Determined from dosing the male animals.

^c^Intravenous dose levels– 1 mg/kg.

^d^Oral dose levels– 2 mg/kg.

AUC, area under the concentration-time curve; CL, clearance; C_max_, maximum plasma drug concentration; ER, extraction ratio; F, bioavailability; PPB, plasma protein binding; P_app_, apparent permeability; t_½_, half-life; V_ss_, steady-state volume or distribution.

In vivo, the systemic clearance of INCB054828 was low in monkeys and dogs (8% and 10% of hepatic blood flow, respectively), but moderate in rats (31% of hepatic blood flow). INCB054828 exhibited a low to moderate volume of distribution in all 3 species, ranging from 0.584 (monkey) to 3.49 L/kg (dog). The terminal elimination half-life following intravenous dosing ranged from 4.0 (rat) to 15.7 hours (dog). The oral bioavailability of INCB054828 was 29% in monkeys, 98% in dogs, and complete in rats. The preclinical pharmacokinetic profile suggests potent inhibition of FGFR1, FGFR2, or FGFR3 is achievable in vivo.

### INCB054828 achieves target inhibition in vivo with low oral doses

**Fig 3:**
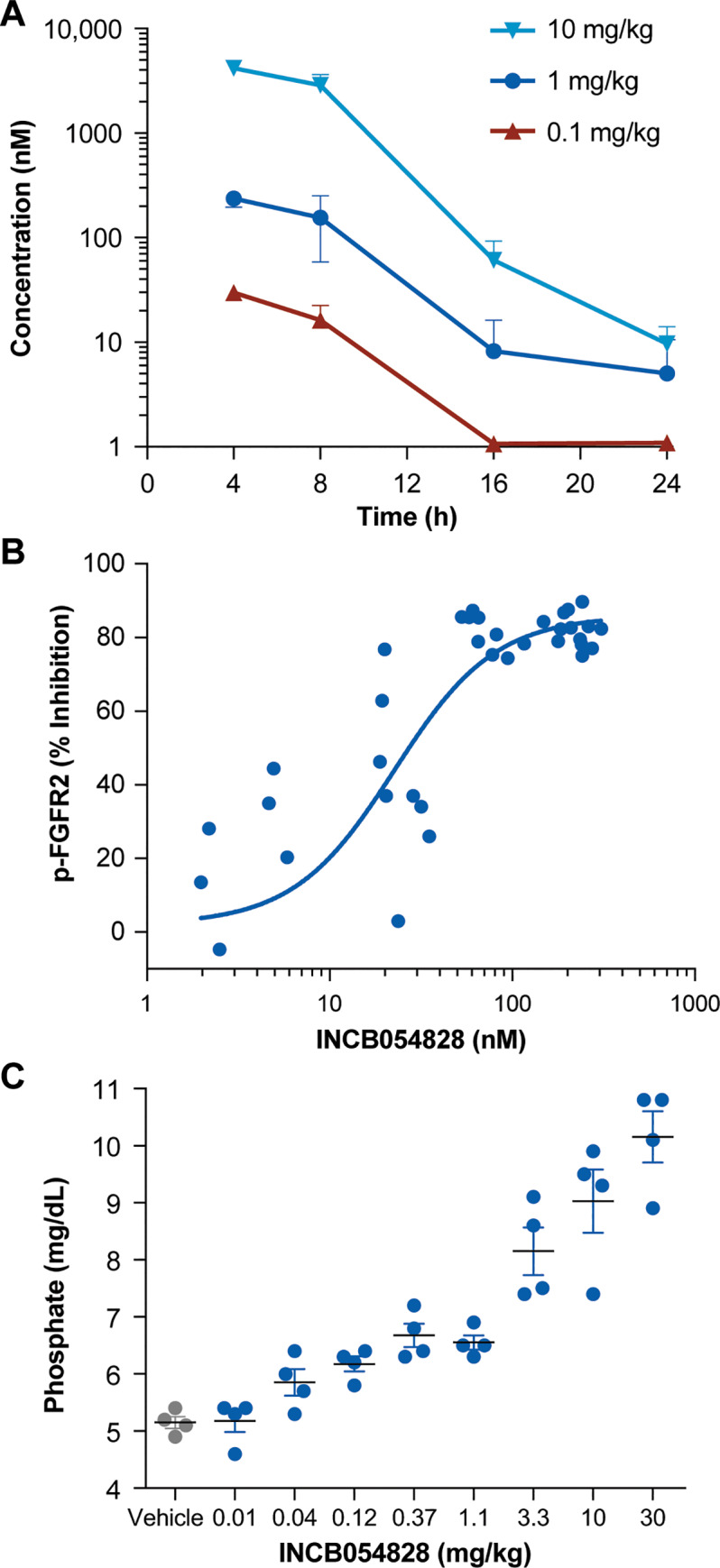
Pharmacokinetics and pharmacodynamics of INCB054828 in the mouse. (A) Pharmacokinetic profile of INCB054828 in the mouse. Plasma was collected over time from mice administered a single oral dose of INCB054828 for determination of total INCB054828 concentration. (B) Pharmacokinetic-pharmacodynamic analyses in KATO III tumors. Tumors and plasma were harvested from KATO III tumor-bearing mice following a single oral administration of INCB054828. Plasma was subjected to analytical analysis and phospho-FGFR2 in tumor homogenate was determined by ELISA. (C) Phosphate levels in the serum of mice following administration of INCB054828. 24 hours after a single oral administration of INCB054828 to C57BL/6 mice, blood was collected and the plasma submitted for analysis of inorganic phosphate.

The plasma exposures after a single oral dose of 0.1, 1, and 10 mg/kg INCB054828 in KATO III tumor-bearing mice were dose linear (Fig 3A). Tumors were harvested for phospho-FGFR2 and analytical analyses, and the exposure-pharmacodynamic curve revealed an in vivo IC_50_ of 22 nM for target inhibition (Fig 3B), similar to the ex vivo-determined spiked KATO III human whole blood value (10.9 nM). Because direct measurement of target engagement in solid tumors could be challenging to assess in patients, a pharmacodynamic assay based on serum phosphate was also investigated. Phosphate levels are regulated by the phosphatonin FGF23 [34]. Inhibition of FGF23 signaling results in phosphorus reabsorption, and changes can be measured in plasma. After a single oral dose of INCB054828, a dose-dependent increase in serum phosphorus was observed in mice (Fig 3C).

### INCB054828 suppresses growth of xenografted tumor models with genetic activation of FGFRs

**Fig 4:**
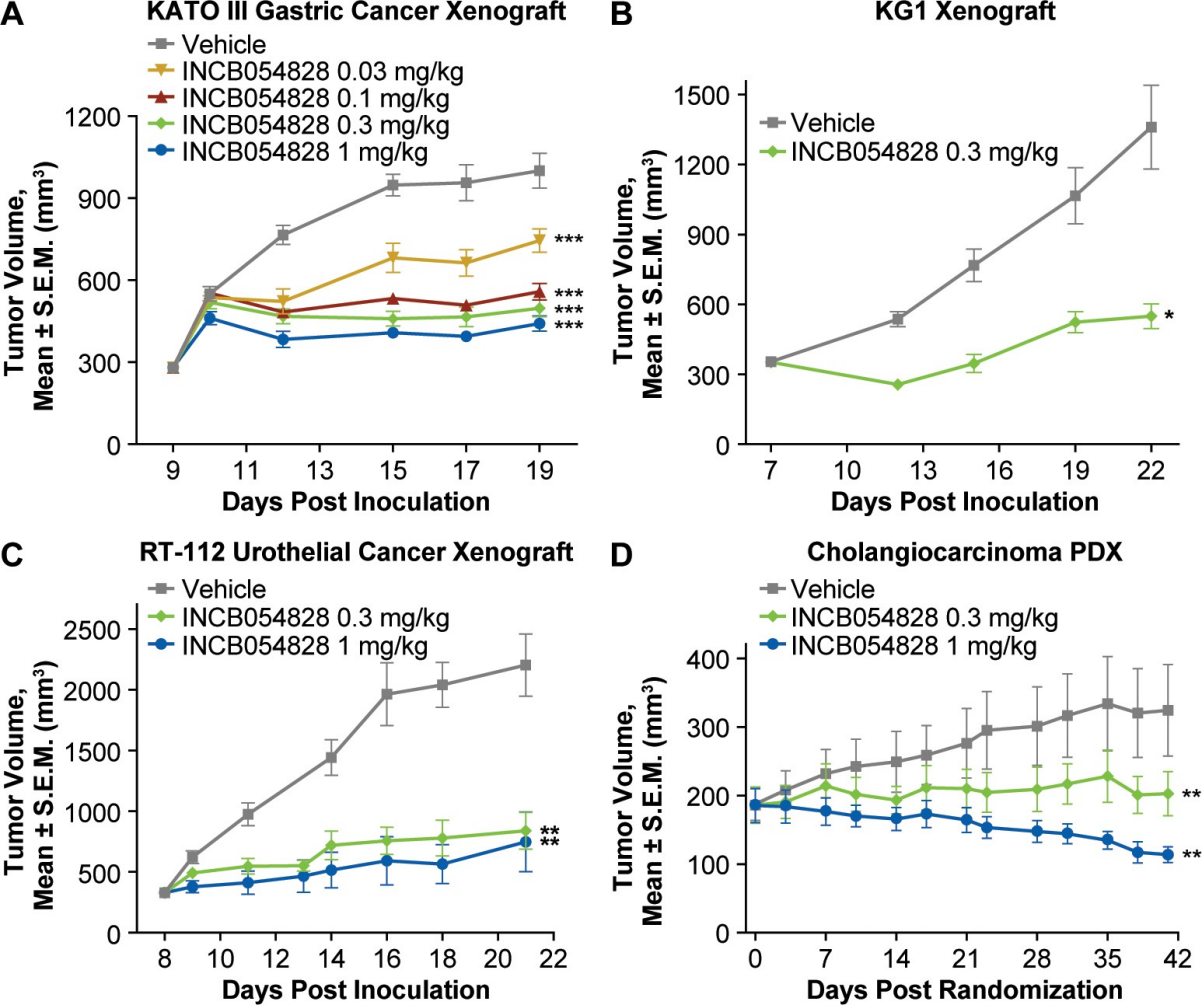
Efficacy of INCB054828 in tumor models with FGFR alterations. (A) KATO III (FGFR2-amplified) gastric cancer model. Severe combined immunodeficiency mice bearing KATO III tumors were administered INCB054828 (0.03, 0.1, 0.3, or 1 mg/kg) or vehicle by gavage once daily for 10 days. The mean tumor size is plotted for each group of 8 mice. ***P < 0.001 vs vehicle. (B) KG1 (*FGFROP2-FGFR1* fusion positive) AML model. Humanized NSG mice bearing KG1 tumors were administered INCB054828 (0.3 mg/kg) or vehicle by gavage once daily for 14 days. The mean tumor size is plotted for each group of 6 mice. *P < 0.05 vs vehicle by paired *t*-test. (C) RT-112 (*FGFR3-TACC3* fusion positive) bladder carcinoma model. RNU immunocompromised rats bearing RT-112 tumors were administered INCB054828 (0.3 or 1 mg/kg) or vehicle by gavage once daily for 14 days. The mean tumor size is plotted for each group of 7 mice. **P < 0.01 vs vehicle. (D) CTG-0997 (*FGFR2-TRA2B* fusion positive) cholangiocarcinoma PDX model. Tumor bearing nu/nu mice were administered INCB054828 (0.3 or 1 mg/kg) or vehicle by gavage once daily for 42 days. The mean tumor size is plotted for each group of 12 mice. **P < 0.01 vs vehicle. S.E.M., standard error of the mean.

The antitumor effect of orally dosed INCB054828 was investigated using xenograft tumor models with genetic alterations in FGFR1 (KG1), FGFR2 (KATO III), and FGFR3 (RT-112). A full dose-response was evaluated using the KATO III gastric cancer model harboring genetic amplification of FGFR2 (Fig 4A). A once-daily dose of 0.03 mg/kg INCB054828 significantly suppressed tumor growth while maximum activity was observed at doses equal to or greater than 0.3 mg/kg once daily. The KG1 erythroleukemia AML cell line carries a translocation of FGFR1 (*FGFROP2-FGFR1)* that has been described in patients with 8p11 myeloproliferative neoplasms. It is the parental line to KG1a, and the in vitro activity of INCB054828 against KG1 and KG1a is similar (GI_50_ values 1 and 3 nM, respectively). A once-daily dose of 0.3 mg/kg showed significant efficacy (*P* < 0.05; Fig 4B) against the KG1 subcutaneous xenograft in a humanized mouse NSG mice engrafted with human CD34+ umbilical cord blood cells. Finally, the activity of INCB054828 was evaluated against an FGFR3-dependent model, RT-112 bladder carcinoma that carries the *FGFR3-TACC3* fusion. This xenograft model was established subcutaneously into nude rats, and oral administration of 0.3 and 1 mg/kg INCB054828 resulted in significant tumor growth inhibition (Fig 4C). Collectively, these data confirm the balanced activity of INCB054828 against FGFR1, 2, and 3 and show that significant efficacy can be achieved with low daily doses. Plasma levels of INCB054828 showed less than 2-fold variation among the xenograft studies at the 1-mg/kg dose for mouse studies.

To extend the translational relevance of these studies further, we identified and used a PDX model of chemorefractory cholangiocarcinoma harboring the *FGFR2-TRA2B* translocation. In mice implanted subcutaneously with these PDX tumors, 10 of 12 tumors showed a reduction in volume with a dose of 1 mg/kg INCB054828, and significant tumor growth inhibition (*P* < 0.01) was observed for both the 0.3- and 1-mg/kg treatment groups compared with vehicle (Fig 4D). In all studies, doses up to and including 1 mg/kg were well tolerated as monitored by a lack of significant body weight changes or other clinical signs compared with the vehicle group including up to 42 days of dosing in the PDX study (S6 Fig).

## Discussion

Deregulation of FGFR signaling is a recurrent event across many cancer types [35]. Preclinical modeling has confirmed that many of these FGFR genetic alterations can be drivers, and growth of cell lines and tumor models bearing such lesions can be effectively inhibited by selective blockade of the aberrant FGFR [8, 9].

In this study, the preclinical data suggest that tumor cells with activated FGF-FGFR signaling can be selectively targeted by the novel FGFR inhibitor INCB054828. This inhibitor is differentiated from many first-generation antiangiogenesis inhibitors because it demonstrates balanced potency against FGFR1, FGFR2, and FGFR3 in biochemical and cellular assays and has high selectivity for these 3 kinases compared with FGFR4 and non-FGFR kinases. This profile translates into potent activity in xenograft tumor models with translocations in FGFR1 (8p11-translocated myeloid leukemia), FGFR2 (cholangiocarcinoma), and FGFR3 (urothelial carcinoma). By sparing FGFR4, effects on bile acid metabolism and potential hepatotoxicity subsequent to bile acid increases may be mitigated [36, 37]. Furthermore, in cell-based assays INCB054828 was more than 80-fold selective against VEGFR, reducing the risk of toxicities associated with inhibition of VEGF signaling that have been observed with first-generation multi-kinase FGFR inhibitors [38]. In preclinical safety studies, liver toxicity and changes in blood pressure were not observed.

In addition to its potency and selectivity, INCB054828 exhibits favorable pharmaceutical properties. INCB054828 exhibited dose-dependent pharmacokinetics in rodents allowing for a thorough characterization of the pharmacokinetic-pharmacodynamic relationship in preclinical models. Pharmacodynamic analyses showed that a plasma target of 22 nM of total INCB054828 was sufficient to inhibit FGFR2 autophosphorylation by 50% in the tumor. At a dose of 1 mg/kg, the plasma exposure of INCB054828 exceeded this IC_50_ target for approximately 12–14 hours per day and was associated with significant efficacy in several tumor models driven by FGFR1, 2, or 3 deregulation. Because a majority of FGFR-dependent cancers are solid tumors, we also investigated the utility of measuring serum phosphate as a surrogate biomarker for FGFR pathway inhibition. As shown previously, inhibition of FGFR signaling antagonizes the phosphaturic function of FGF23 [39], and a dose-dependent increase in plasma phosphorus was observed after a single oral dose of INCB054828. The average half maximal effective dose for phosphate induction from 2 independent experiments was calculated to be 2.9 mg/kg INCB054828, which is higher than the dose required for efficacy in pharmacology studies. Importantly, at doses that were maximally efficacious (0.3–1 mg/kg once daily) in tumor models, no overt toxicities were observed even when INCB054828 was administered for longer than 1 month.

The pharmacokinetic data for INCB054828 from the phase 1 dose-escalation study (NCT02393248) have revealed low clearance and dose-linearity consistent with its preclinical profile [33]. INCB054828 has also demonstrated a favorable safety profile with early signs of clinical activity in several patients with tumors harboring gene fusions with FGFRs. A patient who presented with a myeloid/lymphoid neoplasm with an FGFR1 rearrangement that expressed the fusion *CEP110-FGFR1* transcript achieved a complete cytogenetic and hematologic remission with complete molecular remission of the *CEP110-FGFR1* transcript [40]. The emerging clinical data for other selective FGFR inhibitors have also demonstrated promising activity in several malignancies with FGFR alterations [31, 32, 41]; specifically, erdafitinib, which has been approved for the treatment of patients with urothelial cancers with *FGFR* alterations [42]. Identifying biomarkers for response remains an important translational question, however, as not all cancers with FGFR alterations have shown equivalent responses. These findings suggest that, for example, in lung cancers characterized by FGFR1 gene amplification there is greater tumor complexity or heterogeneity within the amplicon that may modulate the dependency on the FGFR1 [43].

In summary, the preclinical data for INCB054828 together with its preliminary clinical observations suggest that this compound warrants continued investigation in patients selected for FGFR alterations, such as fusions and activating mutations. As a result, phase 2 studies have been initiated in cholangiocarcinoma with FGFR2 fusions (FIGHT 201, NCT02872714), urothelial cancer with activating mutations or fusions (FIGHT 202, NCT02924376), and myeloid/lymphoid neoplasms with FGFR1 rearrangement (FIGHT 203, NCT03011372).

## Supporting information

S1 Appendix Supplemental methods. (DOCX)

S1 Raw Images Original western blot images. (PDF)

S1 Table In Vitro activity of INCB054828 against a panel of Non-FGFR kinases. (DOCX)

S2 Table Percent inhibition data table. (DOCX)

S3 Table Growth inhibition by INCB054828 against cell lines lacking FGFR alterations. (DOCX)

S1 Fig Inhibition of FGFR1 by INCB054828. (DOCX)

S2 Fig A comparison of the cellular potency of INCB054828 for FGFR versus VEGFR directly. (DOCX)

S3 Fig Inhibition of FGFR phosphorylation by INCB054828 in KATO III and Ba/F3 cell lines. (DOCX)

S4 Fig FGFR3 phosphorylation detected by proximity ligation assay. (DOCX)

S5 Fig Effect of INCB054828 on primary T-Cell proliferation. (DOCX)

S6 Fig Mean body weight over time in a CTG-0997 (*FGFR2-TRA2B* Fusion Positive) cholangiocarcinoma PDX model. (DOCX)
