# Supplementary material for: INCB054828 (pemigatinib), a potent and selective inhibitor of fibroblast growth factor receptors 1, 2, and 3, displays activity against genetically defined tumor models
Source: PLoS One. 2020 Apr 21;15(4):e0231877. doi: 10.1371/journal.pone.0231877 (PMC7313537; doi:10.1371/journal.pone.0231877)
Supplement: S1 Appendix — (DOCX) [file pone.0231877.s001.docx]

**INCB054828 (pemigatinib), a potent and selective inhibitor of fibroblast growth factor receptors 1, 2, and 3, displays activity against genetically defined tumor models**

Phillip C.C. Liu^1^, Holly Koblish^1^*, Liangxing Wu^2^, Kevin Bowman^1^, Sharon Diamond^1^, Darlise DiMatteo^1^, Yue Zhang^1^, Michael Hansbury^1^, Mark Rupar^1^, Xiaoming Wen^1^, Paul Collier^1^, Patricia Feldman^1^, Ronald Klabe^1^, Krista A. Burke^1^, Maxim Soloviev^1^, Christine Gardiner^1^, Xin He^1^, Alla Volgina^1^, Maryanne Covington^1^, Bruce Ruggeri^1^, Richard Wynn^1^, Timothy C. Burn^1^, Peggy Scherle^1^, Swamy Yeleswaram^1^, Wenqing Yao^2^, Reid Huber^1^, Gregory Hollis^1^

^1^Discovery Biology, Incyte Research Institute, Wilmington, Delaware, United States of America

^2^Discovery Chemistry, Incyte Research Institute, Wilmington, Delaware, United States of America

^*^Corresponding author

Email: hkoblish@incyte.com; <https://orcid.org/0000-0002-9745-3561>

# Supplemental Methods

## Enzyme selectivity panel

The kinase assays were carried out at room temperature in HEPES 50 mM, pH 7.0, NaN_3_ 0.02%, BSA 0.01%, Orthovanadate 0.1 mM, DTT 1 mM, MgCl_2_ 10 mM in a final volume of 10 µL. Test compounds were transferred to the plate wells by ECHO liquid handler (Labcyte, San Jose, CA) with 0.5% dimethyl sulfoxide (DMSO) in the final assay. Peptide/kinase mixtures were diluted in the appropriate enzymatic buffer and 5µL of the mixture was added to a 384-well small volume black plate. Reactions were initiated by the addition of 5 µL ATP in reaction buffer, the final reaction mixture consisted of 500 nM peptide substrate, 1 mM ATP, and appropriate amount of specific kinase. Reactions were incubated for 90 minutes and terminated by the addition of 10 µL of detection reagent containing 125 nM Streptavidin-XL665, TK (STK) Antibody-Cryptate in HTRF^®^ Detection buffer (HEPES 50 mM, pH 7.0, BSA 0.1%, KF 0.8 M, EDTA 20 mM) (Cisbio, Inc., Bedford, MA). The plates were then sealed and centrifuged at 1800 rpm for 2 minutes. After 60 minutes of incubation at room temperature, the product activity was determined by measuring the fluorescence intensity at 620 nm and 665 nm on Pherastar microplate reader (BMG Labtech, Baden, Germany). Fluorescence intensity ratios were calculated (665 nm/620 nm) for each well; wells with no compound served as the positive controls and those containing no ATP were used as negative controls. Half maximal inhibitory concentration (IC_50_) determination was performed by fitting the curve of percent control activity versus the log of the compound concentration using the GraphPad Prism 6.0 software.

**Receptor tyrosine kinase IC_50_ inhibition assay**

The potency of inhibitors was determined with an enzyme discontinuous assay that measures peptide phosphorylation using Förster Resonance Energy Transfer (FRET) measurements to detect product formation. Compounds were serially diluted in DMSO and a 0.5-µL volume was transferred to the wells of a 384-well plate. Human recombinant fibroblast growth factor receptor (FGFR)1 was generated in-house then modified by dephosphorylating with PTP-1B and column purified. Human recombinant FGFR2, 3, 4, and KDR (alias vascular endothelial growth factor receptor [VEGFR]2) were purchased from Thermo Fisher/LifeTech PV3368 (Waltham, MA), MilliporeSigma 14-464 (Burlington, MA), ThermoFisher/LifeTech P3054, and MilliporeSigma 14-630, respectively. For all isoforms of FGFR and KDR, a 10-µL volume of recombinant enzyme was diluted in assay buffer (50 mM HEPES, 10 mM MgCl_2_, 1 mM EGTA, 0.01% Tween-20, 5 mM DTT, pH 7.5) and added to the appropriate wells. Negative and positive controls (enzyme blank and enzyme with no inhibitor) were included on each plate. The kinases were pre-incubated with/without inhibitor for 5 to 15 minutes at ambient temperature. The assay was initiated by the addition of a 10-µL volume containing both biotinylated peptide substrate (biotin-EQEDEPEGDYFEWLE; custom synthesis; purchased from Genemed Synthesis, Inc, San Antonio, TX) and ATP in assay buffer. The reaction concentration of the peptide substrate was kept constant at 500 nM whereas the ATP concentration was maintained at ATP Km or below for each of the kinases. Hence, the following kinase and ATP concentrations were used: 0.02 nM FGFR1 and 210 µM ATP; 0.01 nM FGFR2 and 100 µM ATP; 0.25 nM FGFR3 and 140 µM ATP; 0.04 nM FGFR4 and 600 µM ATP; 0.15 nM KDR and 1000 µM ATP. The plate was incubated at 25°C for 1 hour and the reactions were ended with the addition of 10 µL/well of quench solution (50 mM Tris, 150 mM NaCl, 0.5 mg/mL BSA, pH 7.8; 30 mM EDTA, 600 nM staurosporine (MilliporeSigma S4400), with Perkin Elmer LANCE^®^ reagents at 3.75 nM Eu-antibody PY20 and 180 nM APC-Streptavidin (Perkin Elmer, Boston, MA). The plate was allowed to equilibrate for ~60 minutes at ambient temperature before scanning the fluorescence intensity (excitation 337 nm/emission 620 nm and 665 nm) on a PheraStar plate reader (BMG Labtech) instrument. The fluorescence intensity ratios were calculated (signals at 665 nm/620 nm x 10^4^) and imported to GraphPad Prism 3.0 software for data analysis. The IC_50_s were derived by fitting the data to a 4-parameter logistic equation producing a sigmoidal dose-response curve with a variable Hill coefficient. Prism equation used: Y=Bottom + (Top-Bottom)/(1+10^((LogIC_50_-X)*Hill slope)) where X is the logarithm of inhibitor concentration and Y is the response.

## Absorption, distribution, metabolism, and excretion assays

***Caco-2 permeability protocol***

The Caco-2 monolayers were incubated at 37°C, with ambient humidity, and CO_2_ for the duration of the transport assay. To determine the apparent permeability of INCB054828 in the absorptive direction (A-B), a 50 μM solution of INCB054828 in Hanks' balanced salt solution was added to the donor compartment (apical side), whereas Hanks' balanced salt solution with 4% BSA was added in the receiver compartment (basolateral side). The apical volume was 0.2 mL and the basolateral volume was 0.6 mL. The incubation period was 120 minutes. At the end of the incubation period, 0.1 mL of sample was removed from the receiver side and an equal volume of acetonitrile was added for protein precipitation. After centrifugation, the supernatants were collected for analysis. Nadolol and metoprolol were included as low and high permeability comparators, respectively. Trans-epithelial electrical resistance was measured to ensure the integrity of the cell monolayers. Caco-2 cell monolayers with trans-epithelial electrical resistance values ≥300 Ω•cm2 were used for transport experiments. The concentrations of the controls and INCB054828 were measured by liquid chromatography/mass spectrometry (LC/MS) and liquid chromatography/tandem mass spectrometry (LC/MS/MS), respectively.

***Intrinsic clearance protocol***

INCB054828 (1 μM) was incubated with human liver microsomes (1 mg/mL of protein), NADPH (2 mM), and 100 mM potassium phosphate buffer (pH 7.4) at 37°C. Aliquots were taken at 0, 10, 20, and 30 minutes and denatured with methanol. After centrifugation to remove the denatured proteins, the resulting supernatants were analyzed by LC/MS

***Protein binding protocol***

The in vitro protein binding of INCB054828 was determined using human, Sprague Dawley rat, and cynomolgus monkey plasma. INCB054828 was prepared as a solution in DMSO and added to plasma to obtain 1-μM substrate concentrations. The final DMSO content in all cases was <0.1%. Each condition was incubated in duplicate. For protein binding determination, the Multi-Equilibrium Dialyzer System™ and diachema membranes from Harvard Apparatus (Holliston, MA) were utilized. Equilibrium dialysis was carried out in Teflon cells separated by a dialysis membrane with a molecular weight cutoff of 10,000 Daltons. A plasma sample (1 mL) containing INCB054828 was added to 1 side of the membrane, while 1 mL of 0.133 M phosphate buffer (pH 7.4) was added to the opposite side. The cells were rotated at a speed of 12 rpm in a 37°C water bath for 2 hours. Following the incubation period, buffer and serum/plasma samples were drained separately into empty preweighed glass test tubes. The tubes were reweighed, and the exact volume of sample recovered from each side of the dialysis cell was calculated using the mass. A 75-μL aliquot of each sample (buffer or serum/plasma) was added to a 96-well plate containing a protein precipitation solution of 100% methanol and 0.2 μM dextrorphan as the internal standard; 75 μL of blank buffer or plasma/serum was added to the plate to ensure identical composition across all samples. Prior to analysis, samples prepared in the 96-well plate were vigorously vortexed for approximately 5 minutes and centrifuged for 10 minutes at ~1700 × g to remove denatured proteins and particulate matter. The supernatant was transferred and analyzed using LC-MS/MS.

***CYP inhibition protocol***

The potential for INCB054828 to inhibit selective substrates for human liver microsomal CYP1A2 (7-ethoxyresorufin), CYP2B6 (bupropion), CYP2C8 (amodiaquine), CYP2C9 (diclofenac), CYP2C19 (S-mephenytoin), CYP2D6 (bufuralol), and CYP3A4 (midazolam or testosterone) was determined using human liver microsomes and a NADPH regenerating system. The final concentrations of INCB054828 were 0, 0.1, 0.25, 1.0, 2.5, 10, and 25 μM. In addition, the known selective inhibitors of CYP1A2 (α-naphthoflavone), CYP2B6 (ticlopidine), CYP2C8 (quercetin), CYP2C9 (sulfaphenazole), CYP2C19 (tranylcypromine), CYP2D6 (quinidine), and CYP3A4 (ketoconazole) were included as positive controls. These assays were conducted at 37°C using a 96-well format with substrates at their respective K_m_ concentrations. Incubations were stopped by the addition of 1 volume of methanol containing 0.2 μM dextrorphan as internal standard. Samples were vortexed and centrifuged, and the resulting supernatants were analyzed using LC-MS/MS.

## Pharmacokinetic analyses

All of our animal studies are done with Institutional Animal Care and Use Committee approval and are in accordance with local, state, and federal guidelines and regulations.

INCB054828 doses were administered intravenously (1 mg/kg) or orally (2 mg/kg) via gavage to Sprague Dawley rats, beagle dogs, and cynomolgus monkeys. The i.v. formulation in rats was 5% dimethylacetamide (DMAC) and 25% sulfobutyl ether β-cyclodextrin in acidified saline (pH 3). The i.v. formulation in dogs and monkeys was 5% DMAC and 20% sulfobutyl ether β-cyclodextrin in saline. The oral formulation in all species was 5% DMAC in 0.5% methylcellulose aqueous solution. For rats, blood samples were collected at predose, 5 (i.v. dosing only), 15, and 30 minutes, and 1, 2, 4, 6, 8, and 24 hours. For dogs, blood samples were collected at predose, 2.5 and 5 (i.v. dosing only), 15, and 30 minutes, and 1, 2, 3, 4, 6, 8, 12, and 24 hours postdose. For monkeys, blood samples were collected at predose, 5 and 10 (i.v. dosing only), 15, and 30 minutes, and 1, 2, 3, 4, 6, 8, 12, 16, and 24 hours postdose. Collected blood samples were processed to extract plasma by centrifugation and stored at −20 ºC until analysis.

The plasma was protein precipitated and the diluted supernatant was analyzed using LC/MS/MS. A standard curve was prepared in plasma from 1 to 5,000 nM and processed in the same manner as the samples. The pharmacokinetic parameters were determined using WinNonLin 6.3.
