# Supplementary material for: INCB054828 (pemigatinib), a potent and selective inhibitor of fibroblast growth factor receptors 1, 2, and 3, displays activity against genetically defined tumor models
Source: PLoS One. 2020 Apr 21;15(4):e0231877. doi: 10.1371/journal.pone.0231877 (PMC7313537; doi:10.1371/journal.pone.0231877)
Supplement: S1 Raw Images — (PDF) [file pone.0231877.s002.pdf]

Supporting Information (S1 File)

INCB054828 (pemigatinib), a potent and selective inhibitor of fibroblast growth factor receptors 1, 2, and 3, displays activity against genetically defined tumor models

Phillip C.C. Liu[1], Holly Koblish[1]*, Liangxing Wu[2], Kevin Bowman[1], Sharon Diamond[1], Darlise DiMatteo[1], Yue Zhang[1], Michael Hansbury[1], Mark Rupar[1], Xiaoming Wen[1], Paul Collier[1], Patricia Feldman[1], Ronald Klabe[1], Krista A. Burke[1], Maxim Soloviev[1], Christine Gardiner[1], Xin He[1], Alla Volgina[1], Maryanne Covington[1], Bruce Ruggeri[1], Richard Wynn[1], Timothy C. Burn[1], Peggy Scherle[1], Swamy Yeleswaram[1], Wenqing Yao[2], Reid Huber[1], Gregory Hollis[1]

[1]Discovery Biology, Incyte Research Institute, Wilmington, Delaware, United States of America

[2]Discovery Chemistry, Incyte Research Institute, Wilmington, Delaware, United States of America

[*]Corresponding author

Email: hkoblish@incyte.com (HK); https://orcid.org/0000-0002-9745-3561

Original Western Blot Images

Blots were developed using enhanced chemiluminescence substrate Femto (Thermo Fisher, Waltham, MA; #34094) and imaged with the Azure Biosystems c300 (Dublin, CA).

Figure 2 Panel A: KG1a (FGFR1OP2-FGFR1)

p-FGFR (Y653/Y654)

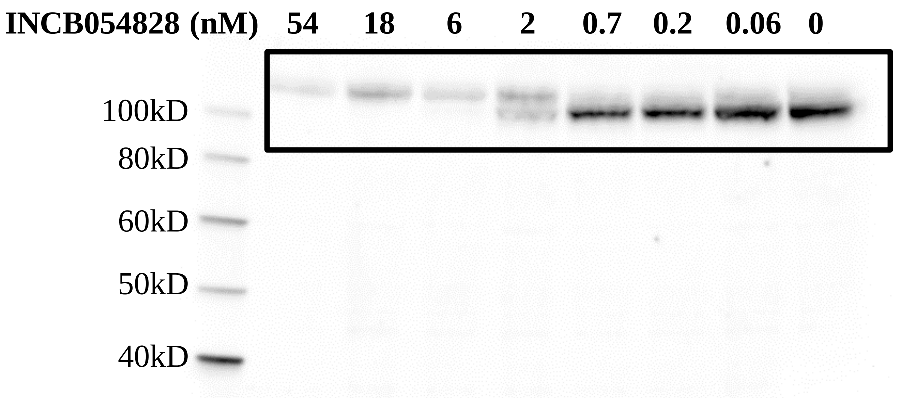

FGFR1

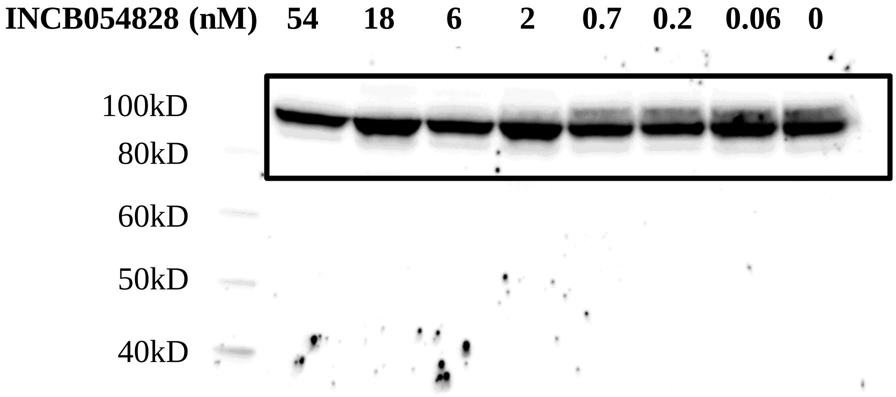

p-ERK1/2 (T202/Y204)

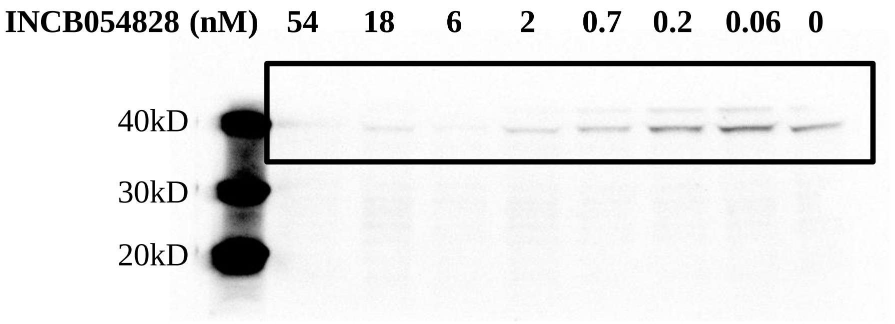

ERK1/2

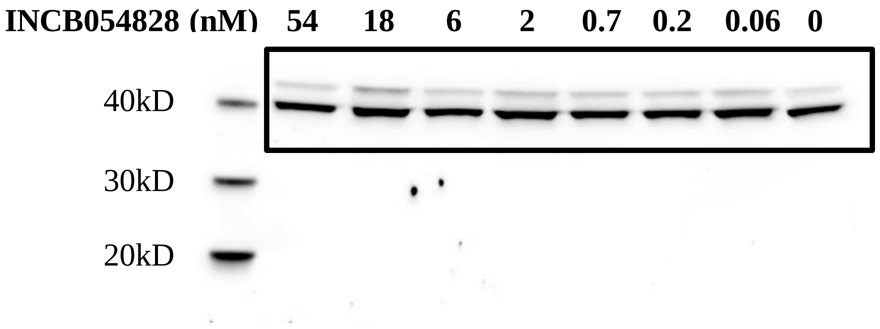

p-STAT5 (Y694)

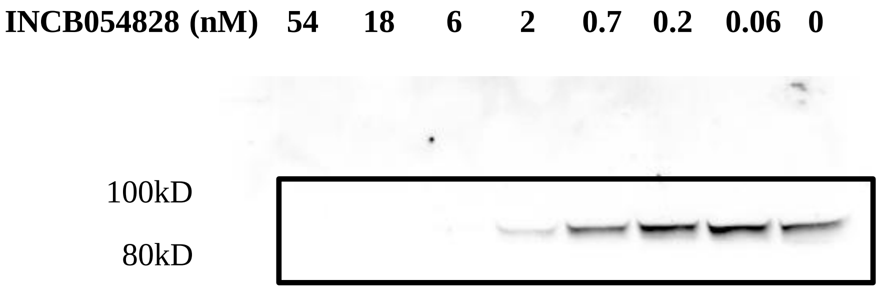

STAT5

INCB054828 (nM) **54** **18** **6** **2** **0.7** **0.2** **0.06** **0**

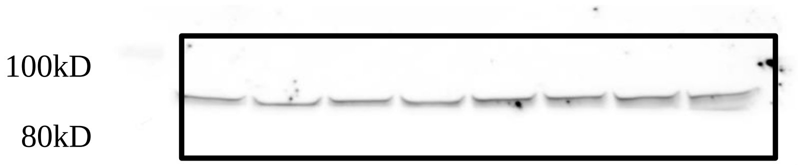

Figure 2 Panel B: RT-4 (*FGFR3-TACC3*)

p-FRS2 (Y436)

INCB054828 (nM) 500 100 25 5 1.25 0.25 0 X

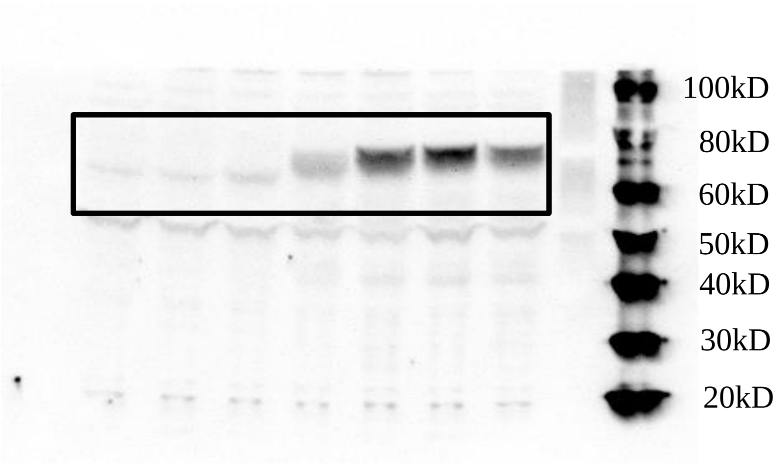

FRS2

INCB054828 (nM) 500 100 25 5 1.25 0.25 0 X

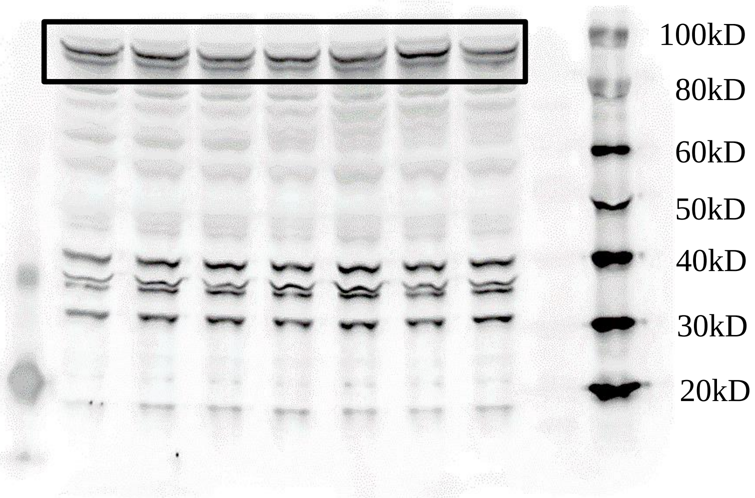

p-ERK1/2 (T202/Y204)

INCB054828 (nM) 500 100 25 5 1.25 0.25 0 X

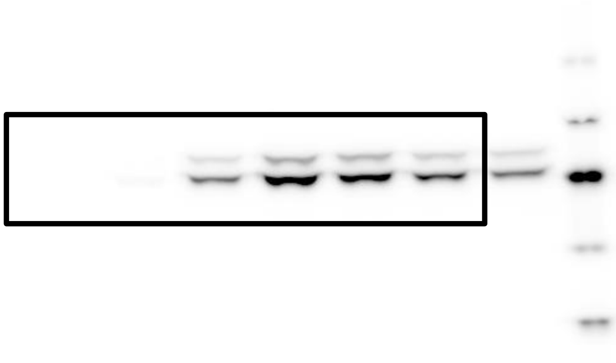

60kD
50kD
40kD
30kD
20kD

ERK1/2

INCB054828 (nM) 500 100 25 5 1.25 0.25 0 X

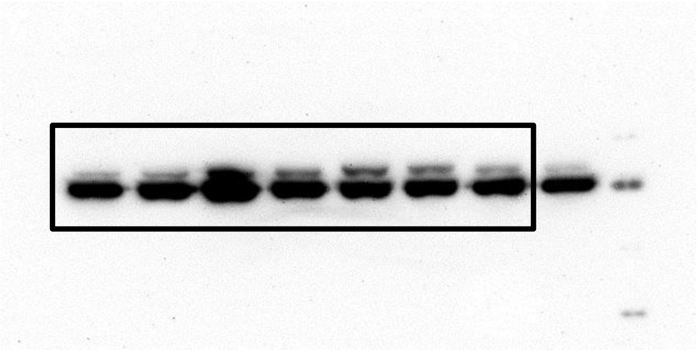

60kD
50kD
40kD
30kD
20kD
