# Supplementary material for: INCB054828 (pemigatinib), a potent and selective inhibitor of fibroblast growth factor receptors 1, 2, and 3, displays activity against genetically defined tumor models
Source: PLoS One. 2020 Apr 21;15(4):e0231877. doi: 10.1371/journal.pone.0231877 (PMC7313537; doi:10.1371/journal.pone.0231877)
Supplement: S1 Table — (DOCX) [file pone.0231877.s003.docx]

**INCB054828 (pemigatinib), a potent and selective inhibitor of fibroblast growth factor receptors 1, 2, and 3, displays activity against genetically defined tumor models**

Phillip C.C. Liu^1^, Holly Koblish^1^*, Liangxing Wu^2^, Kevin Bowman^1^, Sharon Diamond^1^, Darlise DiMatteo^1^, Yue Zhang^1^, Michael Hansbury^1^, Mark Rupar^1^, Xiaoming Wen^1^, Paul Collier^1^, Patricia Feldman^1^, Ronald Klabe^1^, Krista A. Burke^1^, Maxim Soloviev^1^, Christine Gardiner^1^, Xin He^1^, Alla Volgina^1^, Maryanne Covington^1^, Bruce Ruggeri^1^, Richard Wynn^1^, Timothy C. Burn^1^, Peggy Scherle^1^, Swamy Yeleswaram^1^, Wenqing Yao^2^, Reid Huber^1^, Gregory Hollis^1^

^1^Discovery Biology, Incyte Research Institute, Wilmington, Delaware, United States of America

^2^Discovery Chemistry, Incyte Research Institute, Wilmington, Delaware, United States of America

^*^Corresponding author

Email: [hkoblish@incyte.com](mailto:pliu@incyte.com) (HK); <https://orcid.org/0000-0002-9745-3561>

**S1 Table. In Vitro Activity of INCB054828 Against a Panel of Non-FGFR Kinases.**

| **Enzyme** | **IC_50_ (nM)** |
| --- | --- |
| AKT1 | >10,000 |
| AURKA | >10,000 |
| AXL | >10,000 |
| BRAF | >10,000 |
| BTK | >10,000 |
| CAMK2D | >10,000 |
| CDK2 | >10,000 |
| CHEK1 | >10,000 |
| CHEK2 | >10,000 |
| CLK3 | >10,000 |
| DDR2 | >10,000 |
| DYRK2 | >10,000 |
| EGFR | >10,000 |
| EPHA2 | >10,000 |
| EPHB4 | >10,000 |
| ERBB4 | >10,000 |
| FAK | >10,000 |
| FLT3 | >10,000 |
| GSK3B | >10,000 |
| HIPK3 | >10,000 |
| IGF1R | >10,000 |
| IKKA | >10,000 |
| IKKB | >10,000 |
| JNK1 | >10,000 |
| KDR | 182 |
| c-KIT | 266 |
| LCK | >10,000 |
| LOK | >10,000 |
| LRRK2 | >10,000 |
| MAP3K8 | >10,000 |
| MAPKAPK2 | >10,000 |
| MINK | >10,000 |
| MSSK1 | >10,000 |
| NLK | >10,000 |
| PAK4 | >10,000 |
| PDGFR BETA | 1,787 |
| PHKG2 | >10,000 |
| PIM1 | >10,000 |
| PIM2 | >10,000 |
| PKCD | >10,000 |
| PLK3 | >10,000 |
| PRKAA1 | >10,000 |
| RET | >10,000 |
| ROCK1 | >10,000 |
| RPS6KB1 | >10,000 |
| RSK2 | >10,000 |
| SGK1 | >10,000 |
| SGK2 | >10,000 |
| SGK3 | >10,000 |
| SRC | >10,000 |
| STK4 | >10,000 |
| SYK | >10,000 |
| TGFBR1 | >10,000 |
| TIE2 | >10,000 |
| TRKA | >10,000 |
| ZAP70 | >10,000 |
