# Supplementary material for: INCB054828 (pemigatinib), a potent and selective inhibitor of fibroblast growth factor receptors 1, 2, and 3, displays activity against genetically defined tumor models
Source: PLoS One. 2020 Apr 21;15(4):e0231877. doi: 10.1371/journal.pone.0231877 (PMC7313537; doi:10.1371/journal.pone.0231877)
Supplement: S2 Table — (DOCX) [file pone.0231877.s004.docx]

**INCB054828 (pemigatinib), a potent and selective inhibitor of fibroblast growth factor receptors 1, 2, and 3, displays activity against genetically defined tumor models**

Phillip C.C. Liu^1^, Holly Koblish^1^*, Liangxing Wu^2^, Kevin Bowman^1^, Sharon Diamond^1^, Darlise DiMatteo^1^, Yue Zhang^1^, Michael Hansbury^1^, Mark Rupar^1^, Xiaoming Wen^1^, Paul Collier^1^, Patricia Feldman^1^, Ronald Klabe^1^, Krista A. Burke^1^, Maxim Soloviev^1^, Christine Gardiner^1^, Xin He^1^, Alla Volgina^1^, Maryanne Covington^1^, Bruce Ruggeri^1^, Richard Wynn^1^, Timothy C. Burn^1^, Peggy Scherle^1^, Swamy Yeleswaram^1^, Wenqing Yao^2^, Reid Huber^1^, Gregory Hollis^1^

^1^Discovery Biology, Incyte Research Institute, Wilmington, Delaware, United States of America

^2^Discovery Chemistry, Incyte Research Institute, Wilmington, Delaware, United States of America

^*^Corresponding author

Email:[hkoblish@incyte.com](mailto:hkoblish@incyte.com) (HK); <https://orcid.org/0000-0002-9745-3561>

**S2 Table. Percent Inhibition Data Table.**

| **Kinase** | **Average Percent Inhibition INCB054828** |
| --- | --- |
| ABL | -4 |
| Abl (H396P) | -1 |
| Abl (Q252H) | 1 |
| Abl (T315I) | 2 |
| ABL1(E255K) | 6 |
| ABL1(G250E) | 1 |
| ABL1(Y253F) | 2 |
| AKT1 | 2 |
| AKT2 | 2 |
| AKT3 | 3 |
| ALK | -1 |
| AMPK | 4 |
| AMPK-alpha2/beta1/gamma 1 | 7 |
| Arg | -4 |
| AurA | 6 |
| AurB | 11 |
| AurC | -4 |
| AXL | -8 |
| BLK | -10 |
| BMX | 3 |
| BRSK1 | -4 |
| BRSK2 | -2 |
| BTK | -3 |
| Ca MK1a | 3 |
| CamK1d | 14 |
| CAMK2 | 2 |
| Ca MK2a | 2 |
| CAMK4 | -1 |
| Ca MKII_beta | -9 |
| Ca MKII_gamma | -9 |
| Casein kinase 1g2 | -1 |
| CDK1/Cycline B1 | 5 |
| CDK2 | -2 |
| CDK3 | 11 |
| CDK5/p25 | -4 |
| CHK1 | 0 |
| CHK2 | 4 |
| CK1d | -1 |
| CK1-epsilon | -1 |
| CK1g3 (CSNK1G3) | 0 |
| CK1-gamma 1 | -3 |
| CLK2 | -3 |
| c-Raf | 3 |
| CSNK1A1 | -1 |
| c-TAK1 | 2 |
| DAPK1 | 1 |
| DCAMKL1 | -4 |
| DCAMKL2 | 3 |
| DDR2 | -1 |
| DYRK1a | -2 |
| DYRK1B | 7 |
| DYRK3 | 0 |
| DYRK4 | -4 |
| EGFR | 23 |
| EGFR (ErbB1) T790M L858R | 5 |
| EGFR(T790M) | -1 |
| EPHA1 | 2 |
| EPHA2 | 3 |
| EPHA3 | 0 |
| EPHA4 | 3 |
| EPHA5 | 2 |
| EPHA8 | -2 |
| EPHB1 | 0 |
| EPHB2 | 3 |
| EPHB3 | 0 |
| EPHB4 | 4 |
| Erk1 | 1 |
| Erk2 | 3 |
| Fer | 17 |
| FES | -11 |
| FGFR1 | 99 |
| FGFR1 (V561M) | 30 |
| FGFR2 | 98 |
| FGFR2(N549H) | 90 |
| FGFR3 | 98 |
| FGFR3 [K650E] | 84 |
| FGFR4 | 77 |
| FGR | 3 |
| FLT1 | 13 |
| FLT3 | 0 |
| Flt3(D835Y) | 1 |
| FLT4 | 38 |
| FMS | -10 |
| FRK | 0 |
| FYN | -1 |
| GCK | 1 |
| GSK3-alpha | 2 |
| GSK3b | -1 |
| Hck | -3 |
| HER4 | -15 |
| HGK | -6 |
| HIPK1 | -1 |
| HIPK2 | -1 |
| IGF1R | 1 |
| IKBKE (IKK epsilon) | -2 |
| IKK-beta | 4 |
| INSR | 1 |
| IRAK4 | 0 |
| ITK | 2 |
| JAK2 | 8 |
| KDR | 45 |
| KIT | 4 |
| KIT[T670I] | -2 |
| LCK | -2 |
| LOK | -1 |
| LTK | -17 |
| LYN | 14 |
| LYNB | 9 |
| MAPKAPK2 | 0 |
| MAPKAPK3 | 1 |
| MARK1 | 10 |
| MARK2 | 5 |
| MARK4 | -4 |
| MELK | 2 |
| Mer | -1 |
| MET | -2 |
| MET M1250T | 1 |
| MINK | 2 |
| MNK1 (MKNK1) | 6 |
| MSK1 | 1 |
| MSK2 | -7 |
| MST1 | -1 |
| MST1R | 1 |
| MST2 | 3 |
| MST3 (STK24) | 7 |
| NEK1 | 1 |
| NEK2 | -4 |
| NTRK2 (TRKB) | 1 |
| NuaK1 | -3 |
| p38a | 1 |
| p38alpha/SAPK2a (T106M) | -3 |
| p38-beta 2 | 2 |
| p38-delta | 2 |
| p38-gamma | -3 |
| p70S6K | 8 |
| PAK2 | 0 |
| PAK3 | -2 |
| PAK4 | 5 |
| PAK5 (PAK7) | 6 |
| PASK | -7 |
| PDGFR beta | 6 |
| PDGFR_alpha | -4 |
| PDGFRA (D842V) | 18 |
| PDGFR-alpha (V561D) | 1 |
| PhKg1 | -2 |
| PhKg2 | -1 |
| PIM1 | -6 |
| PIM2 | -5 |
| PIM3 | -4 |
| PKA | 1 |
| PKC-alpha | 16 |
| PKCb2 | 0 |
| PKC-beta1 | 7 |
| PKC-delta | 9 |
| PKC-epsilon | 1 |
| PKC-eta | -4 |
| PKC-gamma | 7 |
| PKC-theta | 2 |
| PKCz | 1 |
| PKD1 | -3 |
| PKD2 | -7 |
| PKD3 | 0 |
| PKG1-beta | -2 |
| PKGa | 1 |
| PRAK | -2 |
| PRKCI (PKC-iota) | 3 |
| PRKX | 0 |
| PYK2 | -1 |
| RET | 7 |
| Ret (V804L) | 0 |
| RET Y791F | 6 |
| ROCK1 | -3 |
| ROCK2 | 5 |
| ROS (ROS1) | 3 |
| RSK1 | -2 |
| RSK2 | -2 |
| RSK3 | -3 |
| RSK4 | -1 |
| SGK1 | 1 |
| SGK2 | -2 |
| SGK3 | 13 |
| SRC | 1 |
| SRM (SRMS) | 1 |
| SYK | -2 |
| TEC | 19 |
| TRKC (NTRK3) | 3 |
| TSSK1 | -3 |
| TSSK2 | -3 |
| TXK | -7 |
| TYRO3 | 1 |
| Yes | 3 |
| ZIPK (DAPK3) | 7 |
