# Supplementary material for: INCB054828 (pemigatinib), a potent and selective inhibitor of fibroblast growth factor receptors 1, 2, and 3, displays activity against genetically defined tumor models
Source: PLoS One. 2020 Apr 21;15(4):e0231877. doi: 10.1371/journal.pone.0231877 (PMC7313537; doi:10.1371/journal.pone.0231877)
Supplement: S3 Table — (DOCX) [file pone.0231877.s005.docx]

**INCB054828 (pemigatinib), a potent and selective inhibitor of fibroblast growth factor receptors 1, 2, and 3, displays activity against genetically defined tumor models**

Phillip C.C. Liu^1^, Holly Koblish^1^*, Liangxing Wu^2^, Kevin Bowman^1^, Sharon Diamond^1^, Darlise DiMatteo^1^, Yue Zhang^1^, Michael Hansbury^1^, Mark Rupar^1^, Xiaoming Wen^1^, Paul Collier^1^, Patricia Feldman^1^, Ronald Klabe^1^, Krista A. Burke^1^, Maxim Soloviev^1^, Christine Gardiner^1^, Xin He^1^, Alla Volgina^1^, Maryanne Covington^1^, Bruce Ruggeri^1^, Richard Wynn^1^, Timothy C. Burn^1^, Peggy Scherle^1^, Swamy Yeleswaram^1^, Wenqing Yao^2^, Reid Huber^1^, Gregory Hollis^1^

^1^Discovery Biology, Incyte Research Institute, Wilmington, Delaware, United States of America

^2^Discovery Chemistry, Incyte Research Institute, Wilmington, Delaware, United States of America

^*^Corresponding author

Email: [hkoblish@incyte.com](mailto:pliu@incyte.com) (HK); <https://orcid.org/0000-0002-9745-3561>

**S3 Table. Growth Inhibition by INCB054828 Against Cell Lines Lacking FGFR Alterations.**

| **Cell Line** | **Histology** | **GI_50_ ± S.D. (nM)** |
| --- | --- | --- |
| HCC-422 | Lung cancer | >5,000 |
| A549 | Lung cancer | >5,000 |
| MM1.S | Myeloma | >5,000 |
| KMS12BM | Myeloma | 2,887 ± 524 |
| J82 | Bladder | >5,000 |
| UMUC3 | Bladder | >5,000 |
| HEK293 | Embyronic kidney | >20,000 |
| TF1-BCR-ABL | AML/recombinant | >5,000 |

GI_50_, concentration required to inhibit growth by 50%; S.D., standard deviation.
