# Supplementary material for: INCB054828 (pemigatinib), a potent and selective inhibitor of fibroblast growth factor receptors 1, 2, and 3, displays activity against genetically defined tumor models
Source: PLoS One. 2020 Apr 21;15(4):e0231877. doi: 10.1371/journal.pone.0231877 (PMC7313537; doi:10.1371/journal.pone.0231877)

**INCB054828 (pemigatinib), a potent and selective inhibitor of fibroblast growth factor receptors 1, 2, and 3, displays activity against genetically defined tumor models**

Phillip C.C. Liu^1^, Holly Koblish^1^*, Liangxing Wu^2^, Kevin Bowman^1^, Sharon Diamond^1^, Darlise DiMatteo^1^, Yue Zhang^1^, Michael Hansbury^1^, Mark Rupar^1^, Xiaoming Wen^1^, Paul Collier^1^, Patricia Feldman^1^, Ronald Klabe^1^, Krista A. Burke^1^, Maxim Soloviev^1^, Christine Gardiner^1^, Xin He^1^, Alla Volgina^1^, Maryanne Covington^1^, Bruce Ruggeri^1^, Richard Wynn^1^, Timothy C. Burn^1^, Peggy Scherle^1^, Swamy Yeleswaram^1^, Wenqing Yao^2^, Reid Huber^1^, Gregory Hollis^1^

^1^Discovery Biology, Incyte Research Institute, Wilmington, Delaware, United States of America

^2^Discovery Chemistry, Incyte Research Institute, Wilmington, Delaware, United States of America

^*^Corresponding author

Email:[hkoblish@incyte.com](mailto:hkoblish@incyte.com) (HK); <https://orcid.org/0000-0002-9745-3561>

**S1 Figure. Inhibition of FGFR1 by INCB054828.** (A) Increase in FGFR1 IC_50_ of INCB54828 with increase in ATP concentration. Mean values from duplicates are plotted. (B) Reversibility of phospho-FGFR1/INCB054828 binding (4,000-fold dilution). A 2-hour binding step was performed at high concentrations of 16 nM phospho-FGFR1 combined with 0, 75, and 150 nM INCB054828, followed by a 2-hour dissociation step where samples were diluted 4,000 fold into assay buffer containing INCB054828. Ten µL of diluted samples were added to wells with an equal volume of 420 µM ATP/1,000 nM peptide substrate mix. The wells were incubated for 1 hour at 25°C and the reaction was then quenched with HTRF reagents. Mean values from sextuplicates are plotted.


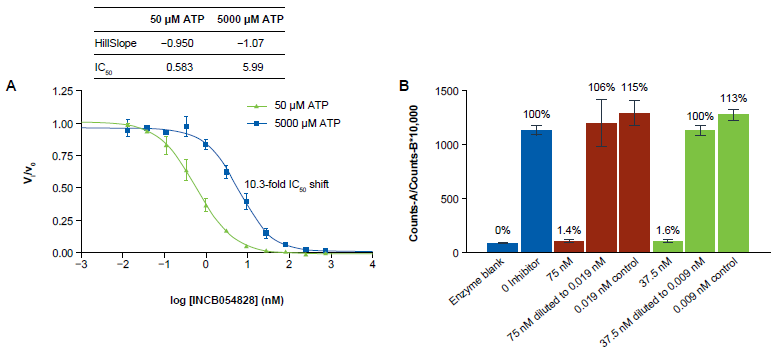

Supplement: S1 Fig — (DOCX) [file pone.0231877.s006.docx]
