# Supplementary material for: INCB054828 (pemigatinib), a potent and selective inhibitor of fibroblast growth factor receptors 1, 2, and 3, displays activity against genetically defined tumor models
Source: PLoS One. 2020 Apr 21;15(4):e0231877. doi: 10.1371/journal.pone.0231877 (PMC7313537; doi:10.1371/journal.pone.0231877)

**INCB054828 (pemigatinib), a potent and selective inhibitor of fibroblast growth factor receptors 1, 2, and 3, displays activity against genetically defined tumor models**

Phillip C.C. Liu^1^, Holly Koblish^1^*, Liangxing Wu^2^, Kevin Bowman^1^, Sharon Diamond^1^, Darlise DiMatteo^1^, Yue Zhang^1^, Michael Hansbury^1^, Mark Rupar^1^, Xiaoming Wen^1^, Paul Collier^1^, Patricia Feldman^1^, Ronald Klabe^1^, Krista A. Burke^1^, Maxim Soloviev^1^, Christine Gardiner^1^, Xin He^1^, Alla Volgina^1^, Maryanne Covington^1^, Bruce Ruggeri^1^, Richard Wynn^1^, Timothy C. Burn^1^, Peggy Scherle^1^, Swamy Yeleswaram^1^, Wenqing Yao^2^, Reid Huber^1^, Gregory Hollis^1^

^1^Discovery Biology, Incyte Research Institute, Wilmington, Delaware, United States of America

^2^Discovery Chemistry, Incyte Research Institute, Wilmington, Delaware, United States of America

^*^Corresponding author

Email:[hkoblish@incyte.com](mailto:hkoblish@incyte.com) (HK); <https://orcid.org/0000-0002-9745-3561>

**S3 Figure.** **Inhibition of FGFR phosphorylation by INCB054828 in KATO III and Ba/F3 Cell Lines.** (A) Assessment of phospho-FGFR2 in the KATO III cell line that expresses high levels of wild-type FGFR2. One representative from 10 assays is shown. (B) The KATO III cell line was spiked into normal donor blood with serial dilutions of the inhibitor to correct for human protein binding. Results are shown as the mean from 11 experiments. Assessment of the ability of INCB054828 to inhibit autophosphorylation of (C) FGFR1 and (D) FGFR3 in Ba/F3 cell lines. Ba/F3 cells were engineered to express the FGFR kinase domains as fusions with the dimerization domain of ETV6 (TEL). Results are shown as the mean from 3 experiments.


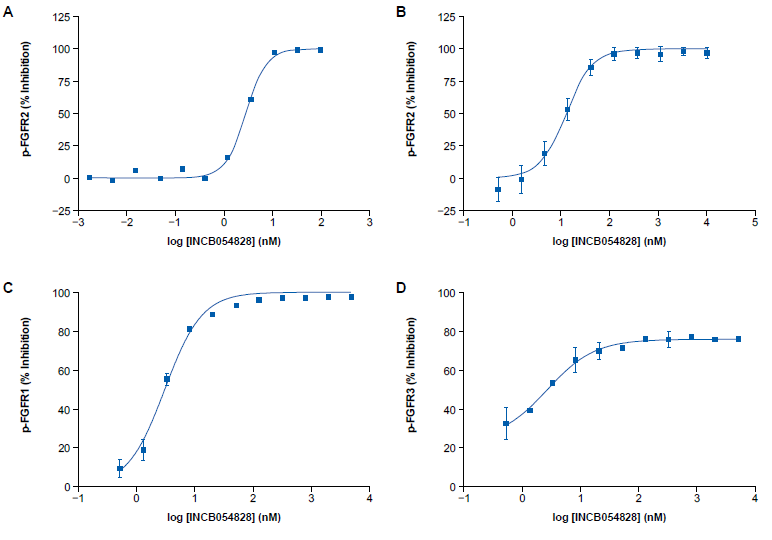

Supplement: S3 Fig — (DOCX) [file pone.0231877.s008.docx]
