# Supplementary material for: INCB054828 (pemigatinib), a potent and selective inhibitor of fibroblast growth factor receptors 1, 2, and 3, displays activity against genetically defined tumor models
Source: PLoS One. 2020 Apr 21;15(4):e0231877. doi: 10.1371/journal.pone.0231877 (PMC7313537; doi:10.1371/journal.pone.0231877)

**INCB054828 (pemigatinib), a potent and selective inhibitor of fibroblast growth factor receptors 1, 2, and 3, displays activity against genetically defined tumor models**

Phillip C.C. Liu^1^, Holly Koblish^1^*, Liangxing Wu^2^, Kevin Bowman^1^, Sharon Diamond^1^, Darlise DiMatteo^1^, Yue Zhang^1^, Michael Hansbury^1^, Mark Rupar^1^, Xiaoming Wen^1^, Paul Collier^1^, Patricia Feldman^1^, Ronald Klabe^1^, Krista A. Burke^1^, Maxim Soloviev^1^, Christine Gardiner^1^, Xin He^1^, Alla Volgina^1^, Maryanne Covington^1^, Bruce Ruggeri^1^, Richard Wynn^1^, Timothy C. Burn^1^, Peggy Scherle^1^, Swamy Yeleswaram^1^, Wenqing Yao^2^, Reid Huber^1^, Gregory Hollis^1^

^1^Discovery Biology, Incyte Research Institute, Wilmington, Delaware, United States of America

^2^Discovery Chemistry, Incyte Research Institute, Wilmington, Delaware, United States of America

^*^Corresponding author

Email:[hkoblish@incyte.com](mailto:hkoblish@incyte.com) (HK); <https://orcid.org/0000-0002-9745-3561>

**S4 Figure. FGFR3 Phosphorylation Detected by Proximity Ligation Assay.** RT-4 cells were treated with 100 nM INCB054828 for 1 hour. Primary antibodies were rabbit anti-FGFR3 (Abcam, Cambridge, UK, #137084) at a 1:250 dilution and mouse 4G10 platinum anti-phosphotyrosine monoclonal cocktail IgG2b (Millipore, Burlington, MA, #05-1050) at a 1:250 dilution. Secondary probes were Duolink In Situ PLA^®^ Probe Anti-Rabbit PLUS, affinity purified donkey anti-rabbit IgG (H+L) and Duolink In Situ PLA^®^ Probe Anti-Mouse MINUS, Affinity purified donkey anti-mouse IgG (H+L). Detection was via Duolink In Situ Detection Reagents Red.


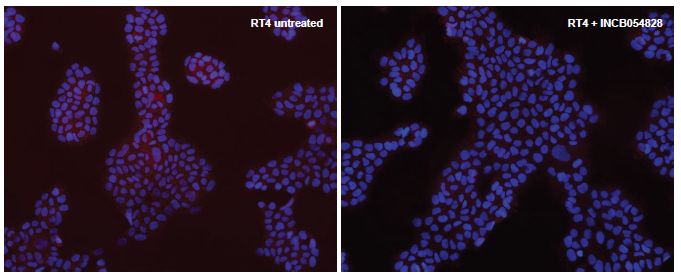

Supplement: S4 Fig — (DOCX) [file pone.0231877.s009.docx]
